# Evolutionarily conserved mechanisms regulating stress-induced neutrophil redistribution in fish

**DOI:** 10.3389/fimmu.2024.1330995

**Published:** 2024-03-07

**Authors:** Katarzyna Klak, Magdalena Maciuszek, Lukasz Pijanowski, Magdalena Marcinkowska, Joanna Homa, B. M. Lidy Verburg-van Kemenade, Krzysztof Rakus, Magdalena Chadzinska

**Affiliations:** ^1^ Department of Evolutionary Immunology, Institute of Zoology and Biomedical Research, Faculty of Biology, Jagiellonian University, Krakow, Poland; ^2^ Doctoral School of Exact and Natural Sciences, Jagiellonian University, Krakow, Poland; ^3^ Cell Biology and Immunology Group, Department of Animal Sciences, Wageningen University, Wageningen, Netherlands

**Keywords:** stress, neutrophil redistribution, cortisol, CXC chemokines, CXC receptors, GCSFR, MMP9

## Abstract

**Introduction:**

Stress may pose a serious challenge to immune homeostasis. Stress however also may prepare the immune system for challenges such as wounding or infection, which are likely to happen during a fight or flight stress response.

**Methods:**

In common carp (*Cyprinus carpio* L.) we studied the stress-induced redistribution of neutrophils into circulation, and the expression of genes encoding CXC chemokines known to be involved in the regulation of neutrophil retention (CXCL12) and redistribution (CXCL8), and their receptors (CXCR4 and CXCR1-2, respectively) in blood leukocytes and in the fish hematopoietic organ – the head kidney. The potential involvement of CXC receptors and stress hormone receptors in stress-induced neutrophil redistribution was determined by an *in vivo* study with selective CXCR inhibitors and antagonists of the receptors involved in stress regulation: glucocorticoid/mineralocorticoid receptors (GRs/MRs), adrenergic receptors (ADRs) and the melanocortin 2 receptor (MC2R).

**Results:**

The stress-induced increase of blood neutrophils was accompanied by a neutrophil decrease in the hematopoietic organs. This increase was cortisol-induced and GR-dependent. Moreover, stress upregulated the expression of genes encoding CXCL12 and CXCL8 chemokines, their receptors, and the receptor for granulocytes colony-stimulation factor (GCSFR) and matrix metalloproteinase 9 (MMP9). Blocking of the CXCR4 and CXCR1 and 2 receptors with selective inhibitors inhibited the stress-induced neutrophil redistribution and affected the expression of genes encoding CXC chemokines and CXCRs as well as GCSFR and MMP9.

**Discussion:**

Our data demonstrate that acute stress leads to the mobilization of the immune system, characterized by neutrophilia. CXC chemokines and CXC receptors are involved in this stress-induced redistribution of neutrophils from the hematopoietic tissue into the peripheral blood. This phenomenon is directly regulated by interactions between cortisol and the GR/MR. Considering the pivotal importance of neutrophilic granulocytes in the first line of defense, this knowledge is important for aquaculture, but will also contribute to the mechanisms involved in the stress-induced perturbation in neutrophil redistribution as often observed in clinical practice.

## 1 Introduction

The mechanism of the stress response is evolutionarily well-conserved. In fish, stress activates the brain-sympathetic-chromaffin cell axis (the equivalent of the mammalian brain-sympathetic-adrenal medulla axis, SAM) and the brain-pituitary-interrenal cells of the head kidney axis (HPI, the equivalent of the mammalian hypothalamus-pituitary-adrenal axis, HPA) (1). The key mediators are adrenocorticotropic hormone (ACTH), glucocorticoids (GC), and catecholamines. Upon binding of corticotropin-releasing hormone (CRH) to the pituitary gland CRH receptors, ACTH is released. ACTH binds to the melanocortin type 2 receptor (MC2R) in the head kidney to stimulate cortisol synthesis by the interrenal cells (2). While the significance of ACTH in the fish immune system has received little attention, it has been demonstrated in mammals that ACTH is one of the first hormones to bind to receptors on immune cells. It regulates the immune response and triggers an increase in the cytotoxicity of T-lymphocytes (3). Also, adrenaline regulates the immune response by delivering “danger” signals. Adrenaline is released after sympathetic innervation of the head kidney chromaffin cells has activated the SAM axis (4). The effect of adrenaline on immune cells is mainly mediated by β-adrenoreceptors (β-ADR) and is immune suppressive both *in vitro* and *in vivo* (5, 6).

Under natural conditions fish relatively often experience short periods of acute stress, leading to a temporary disturbance of homeostasis. These acute stress events may last for a few minutes to several hours. In contrast, under conditions of intensive aquaculture, disturbances last for many hours a day for weeks to several months and therefore are classified as chronic stress. The most common stress factors that fish experience in aquaculture are crowding, transport and transshipment, and procedures of grading or vaccination. Chronic stress causes loss of homeostasis, to which adaptation is not possible, or needs long time to adapt. In a situation of chronic stress, the response shifts from adaptive to maladaptive, eventually resulting in impaired reproduction, reduced growth, and decreased disease resistance due to immunosuppression (1, 7, 8).

In contrast to the well-known immunosuppressive and anti-inflammatory effects of glucocorticoids (e.g., 9), in all vertebrate groups, a stress-induced glucocorticoid elevation enhances the release of neutrophils (PMNs, polymorphonuclear leukocytes) into circulation (e.g., 10–14). Moreover, cortisol treatment inhibits neutrophil apoptosis (e.g., 15). As neutrophils are important in the first line of the innate immune defense, a prolonged lifespan will maintain higher numbers in circulation to serve as an adaptive mechanism to fight the extra chance of injury or infection during stress.

In all vertebrates, neutrophil function is strictly dependent on their recruitment. In humans, neutrophils represent 50–70% of the total pool of peripheral blood leukocytes (PBL), whereas in mice they comprise only 10–25% of the PBLs (16). The percentage of PMNs in teleost fish greatly varies during periods of homeostasis: from 4% of circulating leukocytes in common carp (*Cyprinus carpio* L.) (17), 5% in the goldfish (*Carassius auratus* L.) and cichlid (*Cichlasoma dimerus*) (18), up to about 15-20% in Atlantic salmon (*Salmo salar* L.) and rainbow trout (*Oncorhynchus mykiss*) (19), and even as much as 80% in Atlantic cod (*Gadus morhua* L.) (20).

Neutrophils are typically the frontline cells recruited to the focus of the infection or the damage site and they predominate in the rapid initial influx of leukocytes (21). At the inflammatory site, activated neutrophils become powerful killers acting via phagocytosis, degranulation, or formation of neutrophil extracellular traps (NETs) (e.g., 22). Moreover, neutrophils produce potent, factors that are toxic for pathogens, like reactive oxygen species (ROS), enzymes e.g., lysozyme and antimicrobial peptides like bactericidal/permeability-increasing protein (BPI), cathelicidins, and defensins (23). The balance between neutrophil production/release and their clearance safeguards homeostasis (24). In mammals, under normal conditions, continuous neutrophil production during granulopoiesis develops solely in the bone marrow (25). These self-renewing, slowly dividing progenitor cells, express chemokine receptors CXCR2 and CXCR4 and are kept in these niches by interacting with stromal cells like osteoblasts that express CXCL12 (also known as stromal cell-derived factor 1, SDF1). The process of granulopoiesis and neutrophil emigration from the hematopoietic tissues is controlled by granulocyte colony-stimulating factor (G-CSF), that under both homeostatic and inflammatory conditions signals through the G-CSF receptor (G-CSFR) (26). During infection, the level of the G-CSF in the bone marrow increases, and activates the synthesis of proteases, including matrix metalloproteinase 9 (MMP-9). Proteases break down the adhesion molecules that connect hematopoietic stem cells (HSCs) to the stromal cell microenvironment. Moreover, G-CSF causes CXCL12 downregulation on bone marrow stromal cells. This action, along with the disruption of the CXCL12-CXCR4 interaction, results in neutrophil release (27). Live imaging in zebrafish (*Danio rerio*) confirmed that constitutive CXCL12-CXCR4 signaling impairs persistent, directed neutrophil motility, thereby retaining neutrophils in regions of high CXCL12 expression and inducing neutrophil aggregates. Depletion of CXCL12 with morpholino oligonucleotides restores neutrophil mobilization (28).

Neutrophils emerge in the bloodstream as terminally differentiated cells. Their chemotaxis to inflammatory areas is stimulated in response to pathogen- and host-derived chemotactic stimuli including CXC chemokines (29). Both in mammals and in fish, interaction of CXCL8 (also known as interleukin 8, IL-8) with CXCR1 and CXCR2, is critical for the recruitment of neutrophils to the site of inflammation. We and others confirmed that teleost CXCL8 chemokines exhibit a functional homology to mammalian CXCL8, and that all teleost fish possess at least one form of CXCL8 (CXCL8_L1), while two distinct forms of this chemokine can be found in carp and zebrafish (CXCL8_L1 and CXCL8_L2) (30–32). In carp, recombinant CXCL8 molecules from both forms (CXCL8_L1 and L2) induced chemotaxis of neutrophils (30). Moreover, recently we also revealed that in fish, CXC chemokines and their receptors are important regulators of the stress response at multiple levels of the stress axis, with particularly pronounced effects on steroidogenesis and cortisol conversion in the head kidney (33).

Mechanisms regulating stress-induced neutrophil redistribution are less understood. Teleost fish are an especially intriguing model for stress-immune interaction studies because their major hematopoietic organ, the head kidney, combines immune and endocrine functions (e.g., cortisol and catecholamine production), and thus these immune processes may be under direct, paracrine control. More than half of all extant vertebrate species belong to this group (35,000 species). Teleost fish are found in essentially every aquatic habitat and have been successful in adapting to different environments and we postulate that the ability to control and co-ordinate endocrine and immune responses during environmental challenges must have contributed to this success.

We now aimed to reveal evolutionarily conserved mechanisms involved in the regulation of neutrophil redistribution during stress in common carp. We studied the stress-induced redistribution of neutrophils from the head kidney, into the circulation and the gene expression of CXC chemokines (CXCL12 and CXCL8) and their receptors (CXCR4 and CXCR1-2). We also investigated the involvement of CXC receptors and GR/MR, ADR and MC2R, in stress-induced PMN redistribution by an *in vivo* study of the effects of selective inhibitors of CXCR1, 2 and 4 chemokine receptors and GR/MR, ADR and MC2R antagonists during stress in common carp.

## 2 Materials and methods

### 2.1 Animals

Juvenile common carp (*Cyprinus carpio* L. body weight (b.w.)) 70-120 g, 8-10 months; line R3xR8) were obtained from the Institute of Ichthyobiology and Aquaculture, Polish Academy of Science, Golysz, Poland. Prior to the experiments, fish were adapted for 4 weeks at 21°C and 12L:12D light/dark cycle in recirculating tap water at the Institute of Zoology and Biomedical Research in Krakow, Poland. Fish were kept in tanks (volume 375 l, 45 fish/tank) and daily fed with pelleted dry commercial feed (Aller Master, Aller Aqua, Czarna Dabrowka, Poland) at a total ratio of 1% of their estimated b.w. and with the bloodworm (the midge larvae from a Chironomidae family). To avoid additional stress and/or differences in handling, all samplings were performed by the same person and at the same time of day.

In the blood serum of each animal the free cortisol levels were determined using a commercially available Neogen, Lexington Cortisol ELISA Kit (KY, USA) and levels of glucose were measured with an iXell^®^ glucometer (Ganexo, Warsaw, Poland) as described previously by Klak et al. (33).

All animals were handled in strict accordance with good animal practice as defined by the relevant national and local animal welfare bodies, and procedures were approved by the local ethical committee (2nd Local Institutional Animal Care and Use Committee (IACUC) in Krakow, Poland, license number 292/2017 and 246/2021.

### 2.2 Stress model

Restraint stress was given by netting the fish and suspending the nets with the fish in the water of separate tanks as described previously (14, 34). Fish were stressed for 2, 5, 11 (acute stress) and 24 hours (prolonged acute stress) and sampled at 11.00, 13.00, 20.00 and 9.00, respectively. Immediately after the stress, the fish were deeply anesthetized in 0.4 g/L tricaine methane sulfonate (TMS, Sigma-Aldrich, St. Louis, MO, USA) buffered with 0.8 g/L NaHCO_3_ (POCH, Gliwice, Poland). The control group was sacrificed on the same experimental day at 9.00 a.m. We also verified if control/unstressed fish, sampled at different time points of the day (9.00, 11.00, 13.00 and 20.00), differ in the percentage of PMNs present in the head kidney and peripheral blood and found no statistically significant differences (**Supplementary Figure 1**). The fish were not fed during the stress procedure. The experiments were repeated twice independently with 4 fish per group for each time point.

### 2.3 Pretreatment with antagonists and inhibitors

The following antagonists/inhibitors were used: for the glucocorticoid receptors (GR): mifepristone (RU-486) (Sigma-Aldrich, Saint-Louis, MO, USA, 2 mg/kg b.w.), for mineralocorticoid receptors (MR): spironolactone (Sigma-Aldrich, Saint-Louis, MO, USA, 2 mg/kg b.w.), a selective antagonist for β1-ADR: atenolol (Sigma-Aldrich, Saint-Louis, MO, USA, 0.213 mg/kg b.w.), a β2-ADR antagonist: ICI-118,551 (Sigma-Aldrich, Saint-Louis, MO, USA, 0.25 mg/kg b.w.), the MC2R antagonist: GPS1573 (Cambridge Research Biochemicals, Billingham, UK, 1 mg/kg b.w.), the non-competitive allosteric inhibitor of chemokine CXCR1 and CXCR2 receptors: reparixin (Sigma-Aldrich, Saint-Louis, Missouri, USA, 30 mg/kg b.w.) and the nonpeptide CXCR2 receptor inhibitor: SB225002 (Sigma-Aldrich, Saint-Louis, Missouri, USA, 2 mg/kg b.w.). They were resuspended in sterile dimethyl sulfoxide (DMSO, Thermo Fisher Scientific, Massachusetts, USA). The CXCR4 inhibitor: AMD3100 (plerixafor) (Sigma-Aldrich, Saint-Louis, MO, USA, 1 mg/kg b.w.) was resuspended in sterile phosphate-buffered saline (PBS) (270 mOsM). For all antagonists/inhibitors, effectiveness was previously proven (35–40). Experimental fish were assigned randomly to the vehicle (DMSO/PBS, N = 8) and antagonist or inhibitor treatment groups (N = 8) as described previously (33). Prior to injection, all fish were individually weighed to adjust the dose of a particular drug. To minimalize stress and pain, the weighing and the injection procedure was preceded by a short-term anesthesia with a buffered solution of tricaine methanesulfonate (TMS, Sigma-Aldrich, Saint-Louis, MO, USA, 0.2 g/L with the addition of 0.8 g/L NaHCO_3_, (POCH, Gliwice, Poland)). Antagonist/inhibitor or vehicle (DMSO/PBS) intraperitoneal (i.p.) injection was administered 1 h prior to the stress procedure, at 8 a.m. The final volume for i.p. injections was 100 μL per individual fish. After injection, fish from each group were transferred into tanks. One hour post injection half of the fish from each group was kept undisturbed while half was stressed for 11 h as described above. Every experiment was performed independently 2 times per applied antagonist/inhibitor, each at the same time (8 a.m. injections, 9 a.m. confinement (11 h), 8 p.m. sampling) resulting in 16 control (DMSO/PBS, N = 8 or antagonist/inhibitor, N = 8 injected group) and 16 stressed individuals (DMSO/PBS, N = 8 or antagonist/inhibitor, N = 8 injected group).

### 2.4 Head kidney and peripheral blood leukocytes isolation

For PBLs isolation, blood was removed from the caudal vein into a syringe containing 2 mL of RPMI 1640 (Lonza, Basel, Switzerland), adjusted to carp osmolarity of 270 mOsm/kg with distilled water (cRPMI) containing 0.066 mg/mL of heparin (Sigma-Aldrich, St. Louis, MO, USA) and centrifuged for 10 min at 800 rcf at 4°C to obtain the buffy coat. The buffy coat suspended in cRPMI (containing heparin 0.033 mg/mL) was layered on 3 mL Histopaque-1077 (Sigma-Aldrich, St. Louis, MO, USA) and centrifuged at 2000 rcf for 25 min in 4°C without the brake. PBLs were collected, washed two times in cRPMI (10 min at 800 rcf, 4°C) and resuspended in 1 mL of cRPMI. 100 μL of cell suspension was added to 4% paraformaldehyde (Sigma-Aldrich, St. Louis, MO, USA) for subsequent cytometric analysis while the cell pellet was resuspended in 350 μL RL buffer (EURx, Gdansk, Poland) with 1% (v/v) 2-Mercaptoethanol (Sigma-Aldrich, St. Louis, MO, USA) and kept at -80°C for further RNA analyses.

The head kidney is a paired organ. One part of the head kidney was excised and immersed in Fix RNA buffer (EURx, Gdańsk, Poland). All tissues were stored at 4°C until subsequent determination of gene expression. In turn, the second part of the head kidney was used to obtain a suspension of cells from this organ by passing the tissue through a 100 μm nylon mesh with cRPMI and washed once and resuspended in 1 mL of cRPMI. 100 μL of cell suspension was added to 4% paraformaldehyde (Sigma-Aldrich, St. Louis, MO, USA) for subsequent cytometric analysis.

**Figure 1:**
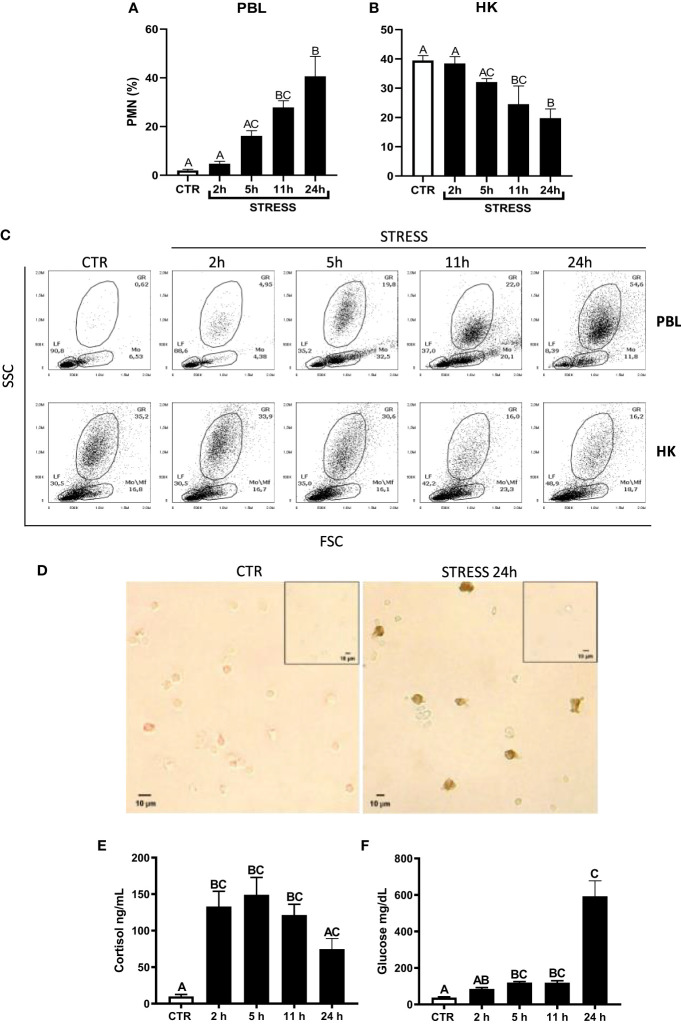
Restraint stress-induced changes in the redistribution of neutrophils (PMN), cortisol and glucose level in common carp. Unstressed control fish and stressed fish were sampled at 2, 5, 11 or 24 h of the experiments. Stress-induced changes in the percentage of PMNs in peripheral blood (PBL, **A**) or in the head kidney (HK, **B**) leukocytes. The percentage of PMNs was measured by flow cytometry in control unstressed fish (CTR, white bars) and in stressed fish (STRESS, black bars) based on cell size (FSC) and granularity (SSC). **(C)** Representative dot plots of PBL and HK from control and stressed fish. **(D)** Representative images of immunocytochemical staining of TCL-BE8 positive cells (granulocytes) in the peripheral blood leukocytes. Negative controls of primary antibody omission are included as figure inserts. **(E)** Cortisol level in serum. **(F)** Glucose level in serum. Data are presented as mean ± standard error (SE) (n ≥ 5). Mean values not sharing letters (e.g., A vs B) indicate statistically significant differences between groups (p ≤ 0.05).

PBLs and cells from HK were measured using a CytoFLEX flow cytometer (Beckman Coulter Inc.). The forward (FSC) and side light scatter (SSC) profiles were adjusted to ensure that all leukocyte populations were clearly displayed. The neutrophil population identified by its typical location was selected by gating (see **Figure 1C**). Counting was terminated when 30,000 leukocytes were counted. Data was analyzed in Microsoft Excel, the sum of P1-P3 gates was taken as 100% leukocytes.

### 2.5 Immunocytochemistry

Cytospins containing 1×10^6^ peripheral blood leukocytes from control and stressed fish after centrifugation on Histopaque-1077 (Sigma-Aldrich, St. Louis, MO, USA) were prepared on glass slides by centrifugation (5 min, 447 x g, at RT; Hettich Universal Tuttlingen, Germany), and then fixed with 4% paraformaldehyde (Sigma Aldrich, St. Louis, MO, USA) and stored at -20°C. Staining was performed as previously described (41). After thawing, cytospin preparations were rehydrated, washed in 0.1 M PBS (pH 7.3), next incubated for 15 min with PBS containing 3% hydrogen peroxide to quench endogenous peroxidase activity, followed by blocking 2% carp inactive serum in a solution containing 1% bovine serum albumin (BSA) (Sigma Aldrich, St. Louis, MO, USA) in PBS at RT for 45 min. Cells were incubated overnight at 4 °C with the primary mouse antibodies TCL-BE8 (1:50) reacting with carp granulocytes (42, 43). For negative control, 1% BSA in PBS was added instead of primary antibodies. After overnight incubation, the slides were rinsed several times in PBS, and then secondary goat anti-mouse IgG antibodies (Bethyl Laboratories.inc, 1:400) were added for 1 h at RT. Then cells were incubated for 1 h with an ExtrAvidyn-peroxidase complex (Sigma-Aldrich, St. Louis, MO) and visualized with 3,3’-diaminobenzidiane (DAB, Sigma-Aldrich, St. Louis, MO, USA). The preparations were analyzed under a light microscope (400 × magnification, Meiji Techno, USA). Pictures were taken with a Moticam10 10.0 MP camera (China). Images were prepared and analyzed in Gimp 2.10. The percentage of TCL-BE8 positive cells in PBLs, obtained from control fish and fish after 24 h of immobilization stress, was calculated. Up to 100 cells were counted on each slide (N = 4).

### 2.6 Gene expression studies

#### 2.6.1 RNA isolation

RNA was isolated with GeneMATRIX Universal RNA Purification Kit (EURx, Gdańsk, Poland) according to the manufacturer’s protocols. Final elution was carried out in 30 μL of nuclease-free water, to maximize the concentration of RNA. RNA quantification and purity were measured by spectrophotometry using a Spark^®^ Multimode Microplate and NanoQuant PlateTM Reader (Tecan, Grödig, Austria). RNA samples were stored at −80°C for further analysis.

#### 2.6.2 cDNA synthesis

The cDNA synthesis reaction was performed using the High-Capacity cDNA Reverse Transcription Kit (Applied Biosystems, Carlsbad, California, USA) following the manufacturer’s protocol. A non-RT (-RT, non-reverse transcriptase) control was included. Samples were diluted 5x and stored at −20°C until further use.

#### 2.6.3 Real-time quantitative PCR

All forward and reverse primer sequences and accession numbers are included in **Supplementary Table 1** (30, 33, 44–47). For RT-qPCR, 4 μL of 50 × diluted cDNA and forward and reverse primers (2 μL each) were added to 7 μL SYBR^®^Select Master Mix (Applied Biosystems, USA). RT-qPCR was initialized for 2 min at 50°C, followed by 2 min at 95°C, and 40 cycles of 15 s at 95°C and 60 s at 60°C, it was performed in a Rotor-Gene Q, 5-Plex HRM (Qiagen, Hilden, Germany). A melting curve analyses were performed at the end of each run. The 40S ribosomal protein s11 (40s11) was used as reference gene. In NTC (non-template control) and -RT control samples, no amplification was observed.

Changes in gene expression upon treatment were determined as a ratio of target gene vs. reference gene (*40s11*) relative to the expression in control samples according to the following equation:


$$\text{Ratio}=\frac{{\left({\text{E}}_{target}\right)}^{\Delta Ct}{\text{Target}}^{\left(control-sample\right)}}{{\left({\text{E}}_{reference}\right)}^{\Delta Ct}{\text{Reference}}^{\left(control-sample\right)}}$$

Where E is amplification efficiency and Ct is the number of PCR cycles needed for the signal to exceed a predetermined threshold value (48).

### 2.7 Statistical analysis

Statistical analysis was performed with GraphPad 9 Software (San Diego, CA, USA). Data were expressed as mean and standard error (SE). Significant differences in all results from experiments verifying stress-induced changes in redistribution of neutrophilic granulocytes as well as changes in the gene expression in control and stressed animals, at different time points, were determined using a one-way analysis of variance (ANOVA) and *post hoc* tests: Tukey’s or Dunn (in case of kinetics of neutrophil redistribution upon stress). The differences were considered statistically significant at p<0.05.

## 3 Results

### 3.1 Stress-induced redistribution of neutrophils

Acute restraint stress induced redistribution of neutrophils from the head kidney to the blood. At 2 and 5 h of stress, the percentage of blood neutrophils slightly and gradually increased, but was not significantly different from the percentages observed in unstressed fish. Starting from 11 h of stress, the percentage of neutrophils in PBLs was significantly higher than in control unstressed animals. At 11 h and 24 h of stress, the percentage of neutrophils in PBLs was at a similar level (**Figures 1A, C**). At 11 h and 24 h of stress, the percentage of neutrophils in the head kidney was lower than in the control unstressed fish (**Figures 1B, C**). Immunocytochemical staining (**Figure 1D**) showed that 24 h of restraint stress increased the number of positive TCL-BE8 cells in PBLs (CTR: 2.82 ± 2.36 vs. STRESS 33.82 ± 14.24; p = 0.0286).

Moreover, stress induced increase in the level of cortisol (**Figure 1E**) and glucose (**Figure 1F**) in serum.

Furthermore, to verify how fast fish will recover post 24 h restraint stress, an additional experiment was performed where upon stress, fish were released from the net for 6 and 12 h (**Supplementary Figure 2**). Already after 6 h of release, the percentage of PMNs in circulation returned to control level. This confirmed that 24 h restraint is a prolonged acute stress and fish are able to recover very fast.

### 3.2 Stress-induced changes in the expression of genes encoding CXC chemokines and receptors, GCSFR and MMP9

**Figure 2:**
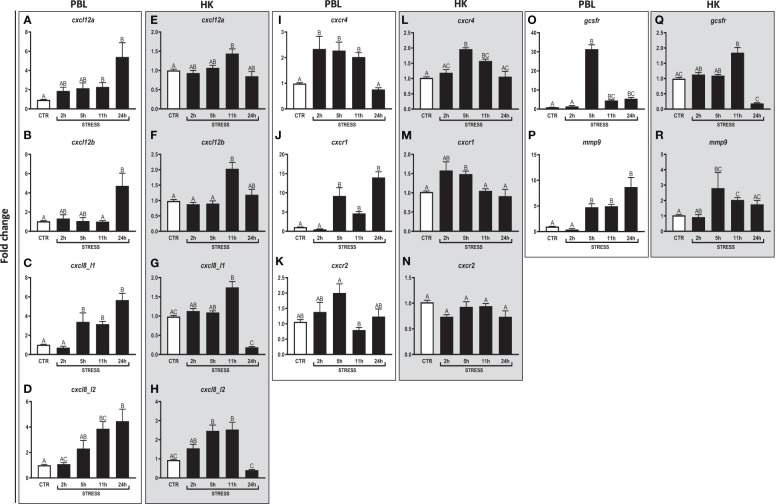
Restraint stress-induced changes in the expression of genes encoding CXC chemokines and receptors, GCSFR and MMP9 in peripheral blood leukocytes (PBL, **A–D**, **I–K**, **O, P**) and in the head kidney (HK, **E–H**, **L–N**, **Q, R**) of common carp. Unstressed control fish (CTR, white bar) and stressed fish (STRESS, black bars) were sampled at 2, 5, 11 or 24 h of the experiments. Gene expression was determined by RT-qPCR and presented as an x-fold increase compared to unstressed fish and standardized for the housekeeping gene 40S ribosomal protein s11. Data are presented as mean ± standard error (SE) (n ≥ 5). Mean values not sharing letters (e.g., A vs B) indicate statistically significant differences between groups (p ≤ 0.05).

In PBLs, upregulation of the expression of *cxcl12a* (**Figure 2A**) and *cxcl12b* (**Figure 2B**) was observed at 11 h and 24 h of stress, respectively, as compared to unstressed animals. Moreover, in PBLs, restraint stress upregulated expression of *cxcl8_l1* (at 5, 11 and 24 h of stress) (**Figure 2C**) and *cxcl8_l2* (at 11 and 24 h of stress) (**Figure 2D**). Stress upregulated also the expression of *cxcr4* (at 2, 5 and 11 h) (**Figure 2I**) and *cxcr1* (at 5, 11 and 24 h) (**Figure 2J**). Furthermore, in PBLs, restraint stress upregulated the expression of *gcsfr* (**Figure 2O**) and *mmp9* (**Figure 2P**) at 5, 11 and 24 h. The highest expression of *gcsfr* was found at 5 h of stress (**Figure 2O**).

In the head kidney, upregulations of *cxcl12a* and *cxcl12b* (**Figures 2E, F**) were demonstrated at 11 h of stress. In this organ, restraint stress also upregulated the expression of *cxcl8_l1* (at 11 h of stress) (**Figure 2G**), *cxcl8_l2* (at 5 and 11 h) (**Figure 2H**), *cxcr4* (at 5 and 11 h) (**Figure 2L**) and *cxcr1* (at 5 h) (**Figure 2M**). In addition, in the head kidney, stress-induced upregulation of *gcsfr* (at 11 h) and *mmp9* (at 5 and 11 h) was also observed (**Figures 2Q, R**).

Both in PBLs and in the head kidney, stress did not affect the expression of *cxcr2* (**Figures 2K, N**).

### 3.3 Effects of hormone receptor blocking on the stress-induced redistribution of neutrophils

**Figure 3:**
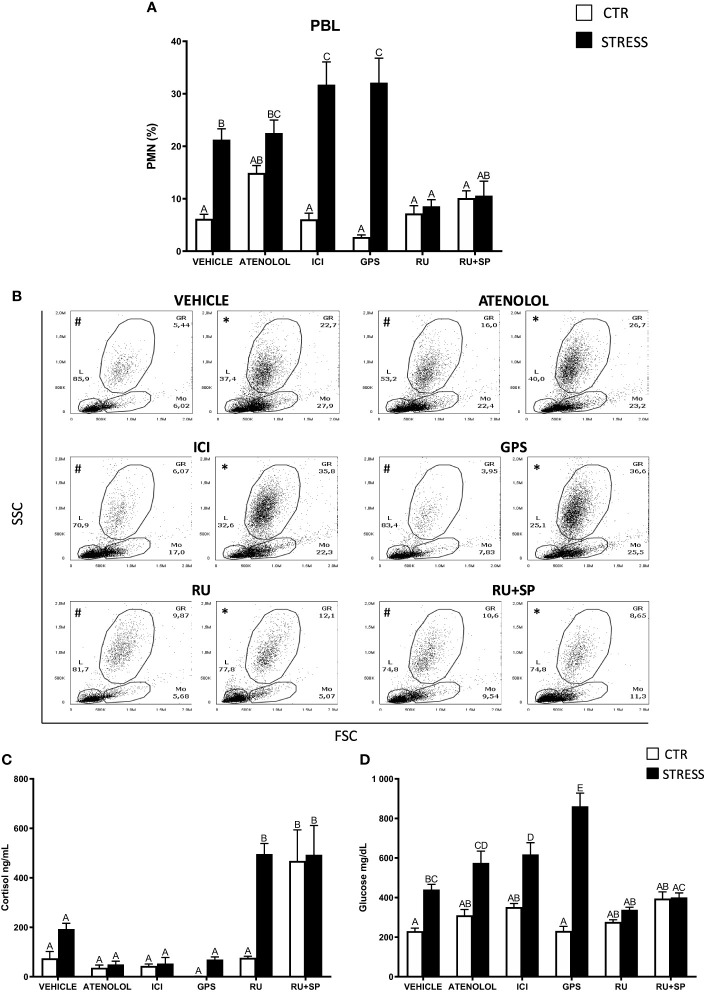
The *in vivo* effects of ADR, MC2R, GR and MR antagonists on changes in neutrophil (PMN) percentage in peripheral blood leukocytes (PBL) and on serum cortisol and glucose level in common carp. 1 hour before stress, fish were i.p. pretreated with: β1-ADR antagonist (atenolol, 0.213 mg/kg b.w.), β2-ADR antagonist (ICI-118,551, ICI, 0.25 mg/kg b.w.), MC2R antagonist (GPS1573, GPS, 1 mg/kg b.w.) or with antagonist of GRs (RU-486, 2 mg/kg b.w.) or with antagonists of GRs and MRs (RU-486 and Spironolactone, RU-486+SP, each 2 mg/kg b.w.). Control animals were treated with vehicle (DMSO). Subsequently, fish were stressed (11 h of restraint, STRESS). Antagonist- or vehicle-treated but unstressed control fish (CTR) were sampled 12 h post-injection. **(A)** The percentage of PMNs was measured by flow cytometry based on cell size (FSC) and granularity (SSC). **(B)** Representative dot plots of PBL from unstressed or stressed (11 h) fish treated with vehicle (VEHICLE) or antagonist (ATENOLOL, ICI, GPS, RU-486, RU-486+SP). # - dot plots from control unstressed fish, * - dot plots from fish stressed 11 h. **(C)** Cortisol level in serum. **(D)** Glucose level in serum. Data are presented as mean ± standard error (SE) (n ≥ 5). Mean values not sharing letters (e.g., A vs B) indicate statistically significant differences between groups (p ≤ 0.05).

To evaluate the involvement of ADR, MC2R, GR and MR receptors in the stress-induced PMN redistribution, antagonists of these receptors were used. 11 h of stress increased the percentage of PMNs in the circulation of vehicle-injected fish, while this reaction was not observed in fish treated with antagonists of GR (RU-486) or GR+MR (RU-486+SP) (**Figures 3A, B**). In contrast, stress-induced increase of the PMN percentage in the blood was not altered in fish treated with the β1-ADR antagonist – atenolol (**Figures 3A, B**). In turn, in stressed fish treated either with β2-ADR (ICI) or MC2R (GPS) antagonist, the PMN percentage in blood was significantly higher than that observed in vehicle-treated stressed animals (**Figures 3A, B**). In the head kidney, the percentage of PMNs decreased after stress challenge and this reaction was not altered upon any of the assessed antagonists, however, the PMN percentage in RU-486-injected unstressed fish was significantly lower than in vehicle-treated unstressed fish (**Supplementary Figure 3**).

In stressed animals that were treated with the GR antagonist, increased levels of serum cortisol were observed compared to stressed fish that were vehicle-treated. Similar high levels of cortisol were also observed in unstressed and stressed fish treated with a combination of GR and MR antagonists (**Figure 3C**). Stressed fish treated with ICI or GPS had higher levels of serum glucose than stressed animals treated with vehicle (**Figure 3D**).

### 3.4 Effects of GR and MR blocking on the expression of genes encoding CXC chemokines and receptors, GCSFR and MMP9

**Figure 4:**
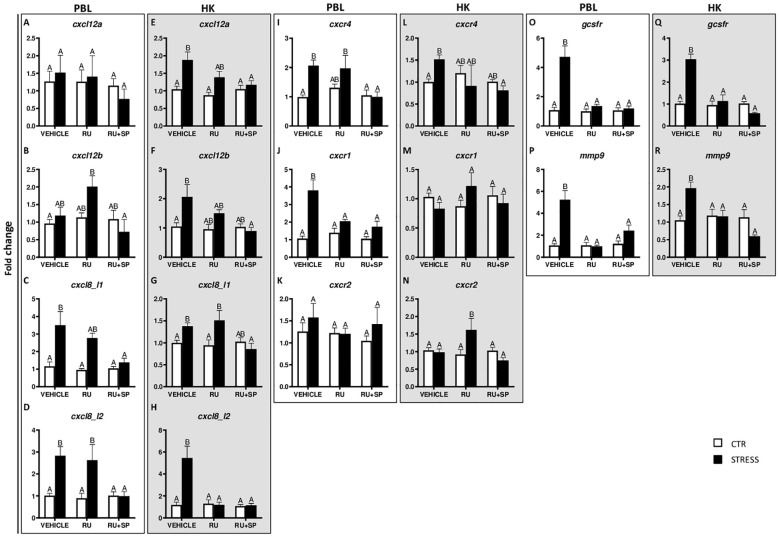
Changes in expression of genes encoding CXC chemokines and receptors, GCSFR and MMP9 in peripheral blood leukocytes (PBL, **A–D**, **I–K**, **O, P**) and in the head kidney (HK, **E–H**, **L–N**, **Q, R**) of common carp. 1 hour before stress, fish were i.p. pretreated with selective antagonists of GR (RU-486, 2 mg/kg b.w.) or GR and MR (RU-486 and spironolactone, RU-486+SP, 2 mg/kg b.w.). Control animals were injected with vehicle (DMSO). Subsequently, fish were stressed (11 h of restraint, STRESS, black bars). Antagonist- or vehicle-treated but unstressed control fish (CTR, white bars) were sampled 12 h post-injection. Gene expression was determined by RT-qPCR and presented as an x-fold increase compared to unstressed fish (CTR) and standardized for the housekeeping gene 40S ribosomal protein s11. Data are presented as mean ± standard error (SE) (n ≥ 5). Mean values not sharing letters (e.g., A vs B) indicate statistically significant differences between groups (p ≤ 0.05).

In PBLs stress induced upregulation of *cxcl8_l1*, *cxcl8_l2* and *cxcr4*, and these effects were not observed in fish treated with RU-486 and spironolactone (RU-486+SP) (**Figures 4C, D, I**). Such upregulation of *cxcr1* was not observed in RU-486- and RU-486+SP-treated groups of stressed fish (**Figure 4J**). In turn, expression of *cxcl12b* was higher in RU-486-treated stressed fish than in RU-486 and spironolactone-treated stressed fish (**Figure 4B**). Neither stress nor antagonist treatment affected the expression of *cxcl12a* and *cxcr2* (**Figures 4A, K**).

In the head kidney, stress induced upregulation of *cxcl12a*, *cxcl12b*, *cxcl8_l1* and *cxcr4* in vehicle-injected fish and this upregulation was not observed in stressed fish treated with RU-486+SP (**Figures 4E–G, L**). Stress-induced upregulation of *cxcl8_l2* expression was not observed in stressed fish treated with RU-486 or RU-486+SP (**Figure 4H**). In the head kidney of stressed fish treated with RU-486, expression of *cxcr2* was higher than in stressed animals treated with vehicle or RU-486+SP (**Figure 4N**). Neither stress nor GR and MR antagonist treatment affected expression of *cxcr1* (**Figure 4M**).

In both PBLs and head kidney, stress-induced upregulations of *gcsfr* (**Figures 4O, Q**) and *mmp9* (**Figures 4P, R**) were not observed in stressed fish treated with RU-486 or RU-486+SP.

### 3.5 Effects of CXCR4 blocking on the stress-induced redistribution of neutrophils

**Figure 5:**
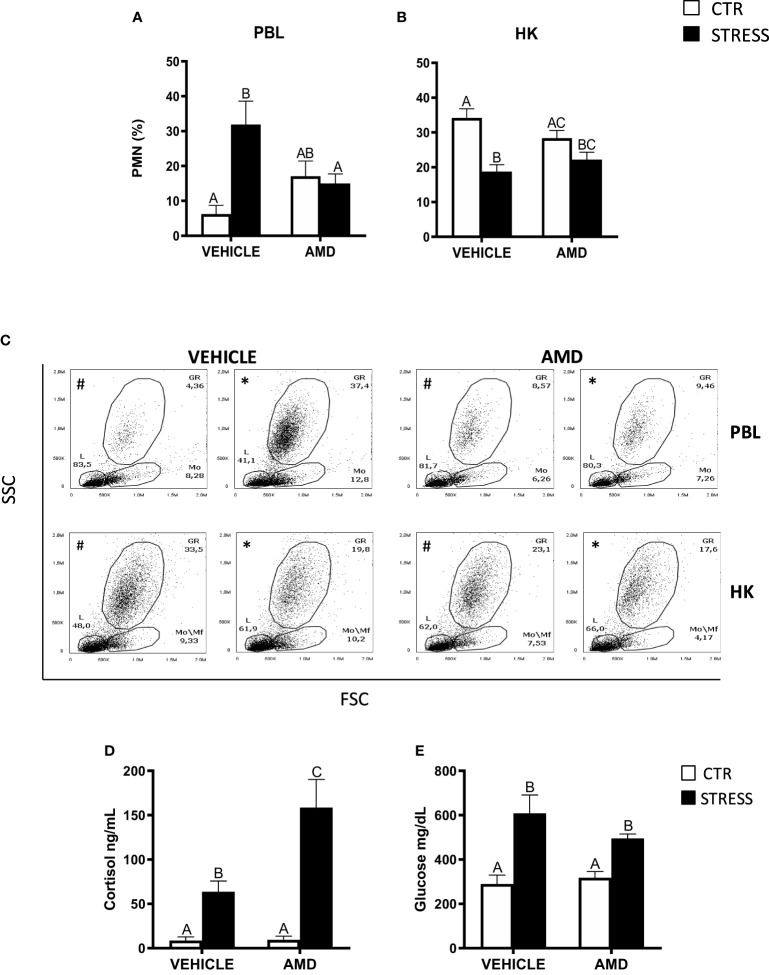
The *in vivo* effect of CXCR4 blocking on the percentage of neutrophils (PMN) in peripheral blood leukocytes (PBL, **A**), in the head kidney (HK, **B**) and on serum cortisol and glucose level in common carp. 1 hour before stress, fish were i.p. pretreated with a selective inhibitor of CXCR4 (AMD3100, AMD, 1 mg/kg b.w.) or with vehicle (PBS). Subsequently, fish were stressed (11 h of restraint, STRESS, black bars). Inhibitor- or vehicle-treated but unstressed control fish (CTR, white bars) were sampled 12 h post-injection. The percentage of PMNs was measured by flow cytometry based on cell size (FSC) and granularity (SSC). **(C)** Representative dot plots of PBL and HK from unstressed and stressed fish treated with vehicle (VEHICLE) or inhibitor (AMD). # - dot plots from control unstressed fish, * - dot plots from fish stressed 11 h. **(D)** Cortisol level in serum. **(E)** Glucose level in serum. Data are presented as mean ± standard error (SE) (n ≥ 4). Mean values not sharing letters (e.g., A vs B) indicate statistically significant differences between groups (p ≤ 0.05).

The stress-induced increase of PMN percentage in the peripheral blood was not observed in stressed fish treated with CXCR4 inhibitor - AMD3100 (**Figures 5A, C**). However, AMD3100 injection did not prevent the stress-induced decrease of the percentage of PMNs in the head kidney (**Figures 5B, C**).

Stressed fish treated with AMD3100 had higher levels of serum cortisol than stressed animals treated with vehicle only (**Figure 5D**), while serum glucose levels were similar in stressed fish that were treated with vehicle or CXCR4 inhibitor (**Figure 5E**).

### 3.6 Effects of CXCR4 blocking on the expression of genes encoding CXC chemokines and receptors, GCSFR and MMP9

**Figure 6:**
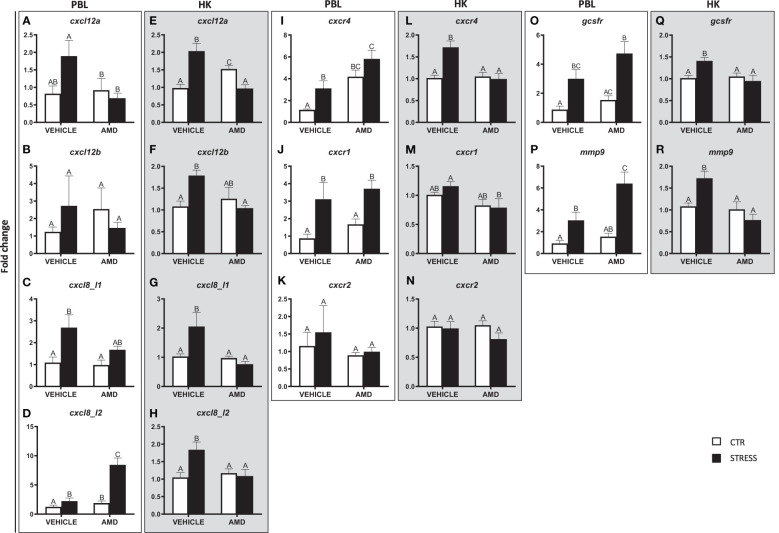
Changes in expression of genes encoding CXC chemokines and receptors, GCSFR and MMP9 in peripheral blood leukocytes (PBL, **A–D**, **I–K**, **O, P**) and in the head kidney (HK, **E–H**, **L–N**, **Q, R**) of common carp. 1 hour before stress, fish were i.p. pretreated with a selective inhibitor of CXCR4 (AMD3100, AMD, 1 mg/kg b.w.) or vehicle (PBS). Subsequently, fish were stressed (11 h of restraint, STRESS, black bars). Inhibitor- or vehicle-treated but unstressed control fish (CTR, white bars) were sampled 12 h post injection. Gene expression was determined by RT-qPCR and presented as an x-fold increase compared to unstressed fish (CTR) and standardized for the housekeeping gene 40S ribosomal protein s11. Data are presented as mean ± standard error (SE) (n = 4-8). Mean values not sharing letters (e.g., A vs B) indicate statistically significant differences between groups (p ≤ 0.05).

In PBLs of stressed fish, AMD3100 treatment decreased the expression of *cxcl12a* (**Figure 6A**). Stress-induced changes in the expression of *cxcl8_l1*, *cxcr1* and *gcsfr* were not prevented by AMD3100 injection (**Figures 6C, J, O**). Moreover, stress-induced upregulation of *cxcl8_l2*, *cxcr4* and *mmp9* in PBLs was enhanced by AMD3100-treatment (**Figures 6D, I, P**). Neither stress nor AMD3100 treatment affected the expression of *cxcl12b* and *cxcr2* in PBLs (**Figures 6B, K**).

In the head kidney, stress-induced upregulations of *cxcl12a*, *cxcl12b*, *cxcl8_l1*, *cxcl8_l2*, *cxcr4*, *gcsfr* and *mmp9* were not observed in AMD3100-treated animals (**Figures 6E–H, L, Q, R**). In the case of *cxcl12a*, in AMD3100-treated unstressed fish, the expression level was higher than in vehicle-treated unstressed fish or in AMD3100-treated stressed fish (**Figure 6E**). In turn, the *cxcr1* expression level in the head kidney of AMD3100-injected stressed fish was lower than in that in vehicle-injected stressed fish (**Figure 6M**). Neither stress nor AMD3100 treatment affected the expression of *cxcr2* in the head kidney (**Figure 6N**).

### 3.7 Effects of CXCR1 and CXCR2 blocking on the stress-induced redistribution of neutrophils

**Figure 7:**
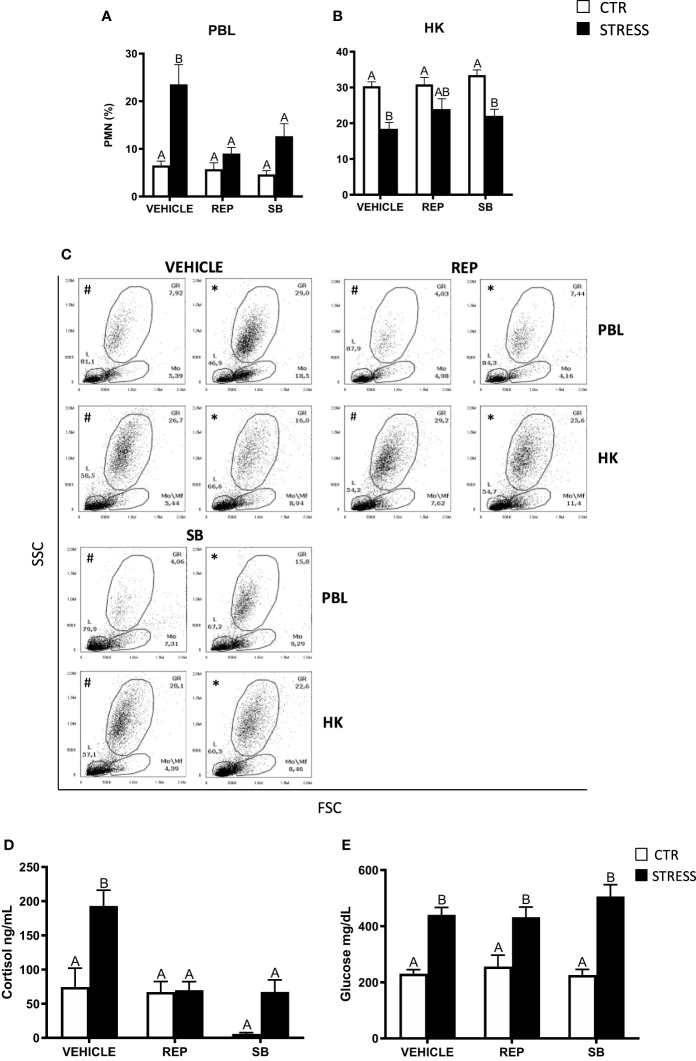
The *in vivo* effects of CXCR1/2 blocking on the percentage of neutrophils (PMN) in peripheral blood leukocytes (PBL, **A**), in the head kidney (HK, **B**) and on serum cortisol and glucose level in common carp. 1 hour before stress, fish were i.p. pretreated with a selective inhibitor of CXCR1 and CXCR2 receptors (Reparixin, REP, 30 mg/kg b.w.), selective CXCR2 receptor inhibitor (SB225002, SB, 2 mg/kg b.w.) or with vehicle (DMSO). Subsequently, fish were stressed (11 h of restraint, STRESS, black bars). Inhibitor- or vehicle-treated but unstressed control fish (CTR, white bars) were sampled 12 h post-injection. The percentage of PMNs was measured by flow cytometry based on cell size (FSC) and granularity (SSC). **(C)** Representative dot plots of PBL and HK from unstressed and stressed fish treated with vehicle (VEHICLE) or inhibitors (REP, SB). # - dot plots from control unstressed fish, * - dot plots from fish stressed 11 h. **(D)** Cortisol level in serum. **(E)** Glucose level in serum. Data are presented as mean ± standard error (SE) (n ≥ 6). Mean values not sharing letters (e.g., A vs B) indicate statistically significant differences between groups (p ≤ 0.05).

The stress-induced increase of the PMN percentage in the peripheral blood was not observed in stressed fish treated with the CXCR1 and CXCR2 inhibitor – reparixin as well as in fish pretreated with the CXCR2 inhibitor - SB225002 (**Figures 7A, C**). However, pretreatment of stressed fish with reparixin or SB225002 had no effect on stress-induced decrease of the PMN percentage in the head kidney (**Figures 7B, C**).

Stress-induced increases of cortisol levels were not observed in animals treated with reparixin or SB225002 (**Figure 7D**), while CXCR1-2 inhibitors did not change the level of serum glucose (**Figure 7E**).

### 3.8 Effects of CXCR1 and CXCR2 blocking on the expression of genes encoding CXC chemokines and receptors, GCSFR and MMP9

**Figure 8:**
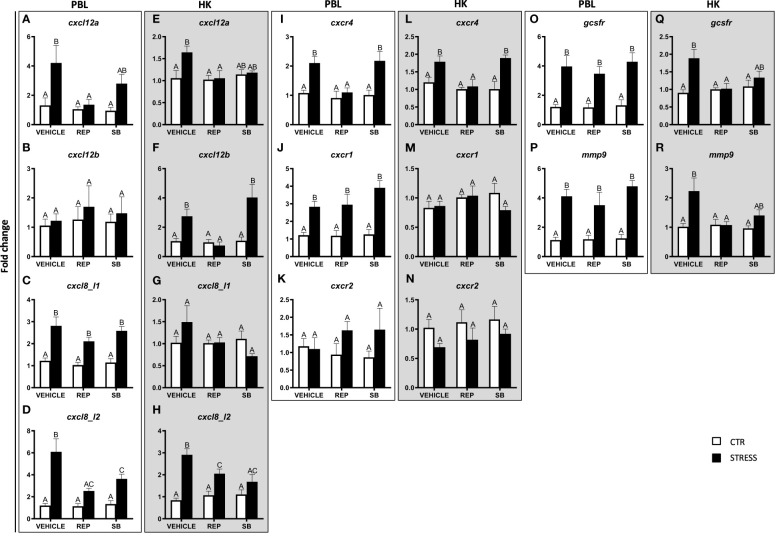
Changes in expression of genes encoding CXC chemokines and receptors, GCSFR and MMP9 in peripheral blood leukocytes (PBL, **A–D**, **I–K**, **O, P**) and in the head kidney (HK, **E–H**, **L–N**, **Q, R**) of common carp. 1 hour before stress, fish were i.p. pretreated with inhibitor of CXCR1 and CXCR2 receptors (Reparixin, REP, 30 mg/kg b.w.), selective CXCR2 receptor inhibitor (SB225002, SB, 2 mg/kg b.w.) or with vehicle (DMSO). Subsequently, fish were stressed (11 h of restraint, STRESS, black bars). Inhibitor- or vehicle-treated but unstressed control fish (CTR, white bars) were sampled 12 h post injection. Gene expression was determined by RT-qPCR and presented as an x-fold increase compared to unstressed fish (CTR) and standardized for the housekeeping gene 40S ribosomal protein s11. Data are presented as mean ± standard error (SE) (n = 5–8). Mean values not sharing letters (e.g., A vs B) indicate statistically significant differences between groups (p ≤ 0.05).

In both PBLs and in the head kidney, expression of *cxcl12a* and *cxcr4* was lower in reparixin-treated stressed fish than in stressed fish treated with vehicle (**Figures 8A, E, I, L**). Similar results were also observed for *cxcl12b*, *gcsfr* and *mmp9* expression in the head kidney (**Figures 8F, Q, R**). Both in PBLs and the head kidney, the expression of genes encoding CXCL12 chemokines and CXCR4 were similar in fish treated with vehicle or with the CXCR2 inhibitor - SB225002 (**Figures 8A, B, E, F, I, L**). Neither stress nor CXCR1/2 inhibitor treatment affected the expression of *cxcl12b* and *cxcr2* in PBLs (**Figures 8B, K**) as well as the expression of *cxcl8_l1*, *cxcr1* and *cxcr2* in the head kidney (**Figures 8G, M, N**).

Moreover, in both PBLs and in the head kidney, stress-induced changes in the expression of *cxcl8_l2* were prevented by reparixin and SB225002 injection (**Figures 8D, H**). In PBLs, stress-induced increases of *cxcl8_l1*, *cxcr1*, *gcsfr* and *mmp9* were modified neither by reparixin nor by SB225002 treatment (**Figures 8C, J, O, P**).

## 4 Discussion

Stress may pose a serious challenge to immune homeostasis, but under certain circumstances, stress may prepare the immune system for challenges such as wounding or infection, which are likely to happen during a fight or flight response (49). Our study focused on the role of stress and GR/MR receptors in the process of neutrophil redistribution in common carp. We hypothesize that the regulation of this phenomenon depends on: (i) neutrophil retention/redistribution signals and/or (ii) cell survival.

Stress induced a time-dependent increase in the level of cortisol and glucose in blood serum (33) and massive elevation of the number of neutrophils circulating in the blood of carp. Our findings are consistent with results in other mammalian and fish models, e.g., in rodents stress hormones induced significant changes in the absolute numbers and relative proportions of white blood cells (12, 50). This precise neuroendocrine tailoring of leukocyte proportions was characterized by the initial mobilization of all leukocytes into the blood (within minutes after the beginning of a stress challenge), followed by a decrease in the number of most white blood cells, while neutrophil numbers, that are key for the innate response, continued to increase (12, 51). Also, Poller et al. (52) showed a rapid neutrophilia triggered by acute stress in mice. The regulation of stress-induced redistribution of neutrophils therefore is an evolutionary conserved phenomenon (10, 53, 54).

Although stress-induced neutrophilia in rats has been linked to noradrenergic signaling (12), Poller et al. (52) have shown that, despite the role of the sympathetic nervous system (SNS) in accelerating hematopoiesis during chronic variable stress, the SNS and specifically adrenergic signaling, does not mediate neutrophilia during acute restraint stress. Also, in our hands blocking of β1-ADR with atenolol or β2-ADR with ICI-118,551 did not reduce the stress-evoked elevation of the number of blood neutrophils. On the contrary, in mice, atenolol, but not ICI-118,551, suppressed neutrophilia induced by acute cold restraint stress (4°C for 1 h) (55). Conflicting reports on the effect of catecholamines on leukocyte redistribution may be caused by the use of different stressors (12). Immobilization, cold stress, or inflammatory pain induced different relative increases in concentrations of catecholamines and glucocorticoids in circulation and this may cause different changes in leukocyte distribution (56, 57). Our study for the first time considers changes in neutrophil redistribution under a prolonged, 11 h restraint stress in fish. Interestingly, we show that, in fish pretreated with the β2-ADR antagonist the number of neutrophilic granulocytes in circulation was even higher than in vehicle-treated stressed fish. This suggests that activation of this receptor inhibits neutrophils release/migration. Previously, an anti-inflammatory action of β2-ADR agonists was observed in rodents and birds. For example, in hens, salmeterol (β2-ADR agonist) reduced the percentage of circulating heterophils (neutrophils of the avian species) compared to control and propranolol (β2-ADR antagonist)-treated birds (58) while in mice isoproterenol (selective β-adrenergic agonist), decreased neutrophil migration during peritonitis (59).

Similarly to β2-ADR-blocking, also the MC2R antagonist increased the number of neutrophilic granulocytes in the circulation of stressed fish above the level observed in vehicle-treated stressed animals. In this case, it cannot be overlooked that GPS1573 retains some antagonist effect on the MSH receptors: MC3R, MC4R and MC5R, but not on MC1R (60). This may, at least partially, explain the GPS-induced upregulation of PMN numbers, as it was previously observed that α-MSH, upon binding to MC4R and MC5R, inhibited the IL-8-induced neutrophil migration *in vitro*, and reduced the neutrophil migration to the site of inflammation *in vivo* (61–63). Moreover, the GPS-induced reduction in cortisol synthesis in stressed fish could be associated with high levels of catecholamines as it is known that the phenylethanolamine-N-methyl transferase (PNMT), which catalyzes the transformation of noradrenaline to adrenaline, is formed only in the presence of high local concentrations of cortisol (64). Therefore, during stress, a low cortisol level potentially elevates the level of noradrenaline. Although, in our hands blocking of β1- and β2-ADRs did not down-regulate the stress-induced neutrophil release into circulation. Dhabhar et al. (12) observed that in adrenalectomized mice, noradrenaline increased the percentage of neutrophils in circulation (2 hpi). Therefore, we can speculate that in stressed fish, in the absence of cortisol, the level of noradrenaline is so high that it stimulates the release of neutrophils.

Usually in mammals, a stress- or infection-induced increase in the number of blood neutrophils reflects a mobilization of cells from different compartments e.g., bone marrow, marginated pool, spleen, lung, or lymph nodes (12). In our studies, a stress-induced increase in blood neutrophils was accompanied by a neutrophil decrease in the hematopoietic organ of fish, the head kidney. Previously, this correlation was also observed in carp after an acute temperature shock (10). This indicates that the head kidney is a source of neutrophils, which emigrate into the circulation during a stress response. However, we cannot exclude that other immunocompetent organ, such as e.g., the trunk kidney or spleen, also serve as a neutrophil reservoir. The latter possibility can be partially supported by our data indicating that, although GR/MR blockade significantly reduced the stress-induced increase of circulating neutrophils, it did not change neutrophil numbers in the head kidney of stressed fish. As cell proliferation and regulated redistribution both take place in the head kidney this is difficult to establish. Our preliminary results indeed show that also in the trunk kidney, stress (11 h and 24 h of restraint) resulted in decreased numbers of neutrophils (**Supplementary Figure 4**). Furthermore, stress-induced changes in the numbers of neutrophils in the blood and in the hematopoietic organs are also influenced by the cortisol-induced and GR-dependent increase in the survival of neutrophils. *In vitro* studies in carp revealed that cortisol rescues neutrophils from apoptosis while RU-486 completely inhibits its anti-apoptotic effect (15). Also *in vivo*, such interaction in the head kidney is feasible as leukocytes, including neutrophils, arise and mature in this organ and they express cortisol receptors (65). At the same time, cortisol is produced in the interrenal cells of the head kidney (9).

Although the mechanisms that regulate neutrophil retention and redistribution during infection/inflammation are well described, little is known about the mechanisms that induce neutrophil motility under stress. Recent elegant work of Tang et al. (50) showed that in mice, acute restraint stress (6 h) induces up-regulation of the inflammatory response in blood leukocytes, suggesting that acute stress leads to an inflammatory state of the body. Therefore, we hypothesize that infection/inflammation- and stress-related mechanisms behind neutrophil mobilization are linked. CXC chemokines and their receptors play a crucial role in neutrophil redistribution upon infection (32, 66–69). During 11 h of stress, both in PBLs and head kidney, we observed upregulation of expression of genes encoding CXCL12s and their receptor CXCR4, as well as the inflammatory CXCL8 chemokines and its putative receptor CXCR1 but not CXCR2. Interestingly, when cortisol receptors were blocked, no such upregulation could be observed, indicating a direct regulation by cortisol-GR/MR interactions.

Both in mammals and zebrafish CXCL12 and CXCR4 interaction plays an important role in neutrophil retention in the hematopoietic tissues (68, 70). However, Walters et al. (28) found that depletion of endogenous CXCL12 mRNA did not affect the neutrophil redistribution in zebrafish larvae, suggesting redundancy with other factors that also mediate neutrophil retention in hematopoietic tissues *in vivo*.

Several studies described the role of CXCL12-CXCR4 interaction during neutrophil migration towards the skin during inflammation and wounding (69–72). For example, in zebrafish larvae, *cxcr4b* and *cxcl12a* were expressed at the wound site, and *cxcr4b* and *cxcl12a* CRISPR knockdown showed that CXCL12/CXCR4 signaling may play an important role in neutrophil retention at inflammatory sites. Both in knockdown larvae and in fish treated with AMD3100, inhibition of the CXL12/CXCR4 signaling accelerated the resolution of the neutrophils from the site of inflammation due to their increased reverse migration (69). Also, Paredes-Zúñiga et al. (70) found in zebrafish larvae that CXCL12a/CXCR4b signaling antagonizes inflammatory signals in response to tissue injury. Compared to wild-type siblings, *cxcr4b* null mutant larvae exhibited increased neutrophil recruitment to wounds. Interestingly, no difference between *cxcr4b* null mutant larvae and their wild type siblings was found in the recruitment of neutrophils to the focus of *S. Typhimurium* infection-induced inflammation.

Upon infection, increased levels of GCSF decrease the level of CXCL12 protein and therefore disrupt CXCL12-CXCR4 interaction and stimulate the release of neutrophils from the bone marrow into the blood (73). In addition, Lévesque et al. (74) found that upon GCSF-induced mobilization of bone marrow progenitor cells, CXCR4 is cleaved and truncated, resulting in the loss of chemotaxis in response to CXCL12. They also found a decrease in the CXCL12 concentration, due to the accumulation of serine proteases (neutrophil elastase and cathepsin G, but not proteinase-3) which directly cleaved and inactivated this chemokine. In our study, GR/MR-dependent up-regulation of expression of genes encoding GCSFR and MMP9 suggests that a similar phenomenon may be initiated during stress in fish. In fish, upregulation of *cxcl12* and *cxcr4* expression was found at 11 h of stress in PBLs and in the head kidney. Petit et al. (75) showed similar results of GCSF-induced transient upregulation of the expression of the CXCL12 gene in bone marrow *in vivo* and *in vitro*, in human and murine osteoblast cell lines. They proved that the gradual decrease of the bone marrow CXCL12 protein is mostly connected with its degradation by neutrophil elastase. As anti-CXCL12 antibodies are unavailable for carp, we cannot check whether, despite the increase in expression at the gene level, a similar or opposite phenomenon also occurs at the protein level. To definitively confirm the importance of the CXCL12-CXCR4 interaction for neutrophil mobilization during stress, we used a selective inhibitor of CXCR4 - AMD3100. In clinical trials, AMD3100 selectively and reversibly antagonized CXCL12 binding to CXCR4, with subsequent egress of hematopoietic stem cells to the peripheral blood (76). In mice, AMD3100 caused a 3-fold increase in the number of circulating neutrophils and this increase corresponded with a decrease of neutrophil numbers in the bone marrow (24). Similar studies in healthy human volunteers revealed that AMD3100 infusion generated a dose-related increase in the number of circulating neutrophils (77). Also, in non-mammalian studies in the little skate (*Leucoraja erinacea*), a significant mobilization of leukocytes upon intraperitoneal administration of AMD3100 was found (78). Surprisingly, at 11 h of stress in AMD3100-treated carp, an increase in the PMN percentage in the circulation was not observed. In unstressed fish treated with AMD3100 the number of neutrophils slightly but not significantly increased in the blood and decreased in the head kidney. During stress, AMD3100 evoked a statistically significant reduction of the neutrophil numbers in the circulation compared to stressed vehicle-treated fish. Moreover, in the head kidney, CXCR4 blockage down-regulated the expression of genes encoding CXCL12s, CXCL8s, MMP-9 as well as CXCR4, CXCR1 and GCSFR, while in PBLs it upregulated expression of *cxcr4*, *cxcl8_l2* and *mmp9*. In this context, it is important to acknowledge that the acute stress response is a critical physiological mechanism, which ensures that leukocytes are present in the right place, at the right time, and in the right state of activation to be prepared for immune challenges that are likely following upon stress under natural conditions (12). Examples of such stress-induced leukocyte trafficking from circulation include skin and other tissues that can be wounded by a predator or stressor (79). Restraint stress by netting, which we used in our study, almost perfectly mimics confinement in nature and involves direct contact of skin with the net. From an evolutionary point of view, such a situation in nature might promote immuno-enhancement of skin tissue, which is now easily exposed to wounding during desperate escape movements. Therefore, we could not exclude that CXCR4 blockage stimulates PMN release at earlier time points of stress, accelerating their migration to injury-exposed organs like the skin. Liu et al. (39) showed that in mice AMD3100 induced the redistribution of lymphocytes, monocytes, and neutrophils from primary immune organs to the secondary immune organs, peripheral tissues and blood, without compromising neutrophil trafficking to inflamed sites. Therefore, we performed an additional experiment and stressed AMD3100-treated fish for 2 h and 5 h. However, in both time points, we did not observe increased neutrophil numbers in the blood circulation (**Supplementary Figure 5**).

Evrard et al. (80) suggested that CXCL12-CXCR4 interaction is crucial mainly for the retention in the hematopoietic tissue of proliferative neutrophil precursors (preNeu), while in non-proliferating neutrophils the expression of CXCR4 is very low. We can therefore hypothesize that these cells are not sensitive to AMD3100. However, in a microbiota-driven process, ageing neutrophils that are present in circulation, upregulate the expression of CXCR4 on their cell surface, which allows them to home back to the bone marrow in response to CXCL12. This ultimately results in the clearance of these leukocytes by resident macrophages (81). Uhl et al. (82) noticed that in mice with endotoxemia, inhibition of the chemokine receptor CXCR4 by AMD3100 or antibody-mediated blockade of CXCL12 did not change the number of aged neutrophils in the circulation and bone marrow. In turn, Devi et al. (83) demonstrated that CXCR4 inhibition via AMD3100 does not result in neutrophil mobilization from the bone marrow. Instead, CXCR4 blockage augmented the frequency of circulating neutrophils through their release from alternative reservoirs/marginated pools present in the lung, while simultaneously preventing neutrophil return to the bone marrow. We therefore postulate that, like during inflammation, also upon stress, “age-wise” cells are the first and dominant subtype of neutrophil to be recruited from hematopoietic tissue. This is in line with the observation that cells, recruited to the circulation during stress, have higher *cxcr4* expression, which is characteristic for aged neutrophils (81). Importantly, although the number of blood neutrophils in AMD3100-treated stressed fish was lower than in stressed animals treated with vehicle, *cxcr4* expression in PBLs from fish with CXCR4 blockage was significantly higher than in fish with “intact” CXCR4.

In mammals, GCSF induces not only loosening of CXCL12-CXCR4 interactions but also increases the CXCL8 concentration in blood circulation. CXCL8, acting via CXCR1 and CXCR2, is one of the most potent neutrophil chemoattractant (84, 85). Through different mechanisms of activation, CXCL8 binding activates these receptors and induces specific intracellular signaling cascades that result in rapid neutrophil recruitment (86–88). Furthermore, Martin et al. (89) found in mice, that maximal mobilization of neutrophils, stimulated by CXCR2-acting chemokines, is dependent on the blockade of the CXCR4-dependent pathway. They also suggested a specific crosstalk between CXCR2 and CXCR4 in which activation of one chemokine receptor evokes desensitization of a second chemokine receptor. Interestingly, during infection/inflammation GCSF also induces granule proteins including MMP-9 (90–92) which further potentiated CXCL8 activity by its N-terminal truncation (90–93).

In carp, stress upregulated the expression of genes encoding CXCL8 chemokines and CXCR1 in both PBLs and head kidney, while expression of the gene encoding CXCR2 remains at the same level in unstressed and stressed fish. In addition, upon stress, a time-dependent upregulation of *mmp9* and *gcsfr* was observed in both PBLs and head kidney. This indicates that CXCL8 chemokines are involved in the regulation of stress-induced neutrophil redistribution from hematopoietic tissues/organs into circulation and likely MMP-9 is involved in their truncation/activation. However, we cannot ignore that MMP-9 is also known for its role in the regulation of neutrophil migration across the basement membrane and the degradation of the extracellular matrix (94).

The role of the interaction between CXCL8 chemokines and their putative receptors CXCR1 and CXCR2 in stress-induced redistribution of neutrophils towards the bloodstream was confirmed in experiments with CXCR1/CXCR2 inhibitors. Both reparixin (inhibitor of CXCR1 and CXCR2) and SB225002 (inhibitor of CXCR2) lowered the number of neutrophilic granulocytes observed in circulation upon stress. Interestingly, only reparixin significantly reduced the stress-induced upregulation of the expression of *cxcl12*, *cxcr4*, *mmp9* and *gcsfr*, while both reparixin and SB225002 prevented such upregulation of *cxcl8_l2*. This suggests that expression of *cxcl12* and its receptor is mainly mediated by CXCR1 while expression of *cxcl8_l1* is regulated by CXCR2 or both CXCR1 and CXCR2.

Interestingly, inhibitors of CXCR4 and CXCR1-2 differentially affected the cortisol levels in stressed fish. We previously published a detailed explanation of the interaction between CXC chemokines and the activation of the stress axis and cortisol synthesis and conversion (33).

Altogether, our data strongly suggest that acute stress led to the mobilization of the immune system, characterized by neutrophilia, and this phenomenon is evolutionary well-conserved. We also revealed that in fish, CXC chemokines and their receptors are involved in the stress-induced redistribution of PMNs from the hematopoietic tissue into the peripheral blood and that this phenomenon is directly regulated by interactions between cortisol and the GR/MR.

Considering the pivotal importance of neutrophilic granulocytes to the first line of defense, our results will not only be important for aquaculture, but they will also contribute to revealing the mechanisms involved in the stress-induced perturbation in neutrophil redistribution as often observed in clinical practice. Prominent examples are Cushing disease, characterized by increased secretion of ACTH, or glucocorticoid resistance/insensitivity of several common inflammatory diseases.

## Data availability statement

The datasets presented in this study can be found in online repositories. The names of the repository/repositories and accession number(s) can be found below: https://doi.org/10.57903/UJ/GM6MQF, 10.57903/UJ/GM6MQF.

## Ethics statement

The animal study was approved by 2nd Local Institutional Animal Care and Use Committee (IACUC) in Krakow, Poland, license number 292/2017 and 246/2021. The study was conducted in accordance with the local legislation and institutional requirements.

## Author contributions

KK: Writing – review & editing, Data curation, Formal analysis, Investigation, Methodology, Writing – original draft. MMac: Data curation, Formal analysis, Investigation, Methodology, Writing – original draft, Writing – review & editing. LP: Investigation, Methodology, Writing – review & editing. MMar: Investigation, Writing – review & editing. JH: Investigation, Methodology, Writing – review & editing. BMLVvK: Conceptualization, Writing – review & editing, Data curation. KR: Conceptualization, Writing – review & editing. MC: Conceptualization, Data curation, Formal analysis, Funding acquisition, Methodology, Project administration, Supervision, Writing – original draft, Writing – review & editing.
