# Supplementary material for: Evolutionarily conserved mechanisms regulating stress-induced neutrophil redistribution in fish
Source: Front Immunol. 2024 Mar 7;15:1330995. doi: 10.3389/fimmu.2024.1330995 (PMC10954836; doi:10.3389/fimmu.2024.1330995)
Supplement: Supplementary file 1 [file DataSheet_1.pdf]

Supplementary Material

1 Supplementary Tables

Supplementary Table 1. Primers sequences, corresponding accession numbers, concentrations and references.

Gene (Acc.no)	Primer nucleotide sequences (5'-3')	µM	References
40s11 **(AB012087)**	**F:** CCGTGGGTGACATCGTTACA<br>**R:** TCAGGACATTGAACCTCACTGTCT	1	Huising et al., 2003, 2004; Chadzinska et al., 2008; van der Aa et al., 2010, 2012; Klak et al., 2022
cxcl12a **(AJ627274)**	**F:** CACCGTCACAGATATGTACCATATAGTC<br>**R:** GGTGGTCTTTTGCAGAGTCATTT	1	Huising et al., 2004; Klak et al., 2022
cxcl12b **(AJ536027.3)**	**F:** GAGGAGGACCACCATGCATCT<br>**R:** TTGTGCAAGCAGTCCAGAAGA	1	Huising et al., 2004; Klak et al., 2022
cxcr4 **(AB012310.1)**	**F:** CGGTGTCTGCGTTCATCTATT<br>**R:** TTCTTTTGGAAGCCCATCAC	1	Klak et al., 2022
cxcl8_l1 **(AJ421443)**	**F:** CTGGGATTCCTGACCATTGGT<br>**R:** GTTGGCTCTCTGTTTCAATGCA	1	Chadzinska et al., 2008, van der Aa et al., 2010, 2012, Klak et al., 2022
cxcl8_l2 **(AB470924)**	**F:** TCACTTCACTGGTGTTGCTC<br>**R:** GGAATTGCTGGCTCTGAATG	1	van der Aa et al., 2010, 2012; Klak et al., 2022
cxcr1 **(AB010468)**	**F:** GCAAATTGGTTAGCCTGGTGA<br>**R:** AGGCGACTCCACTGCACAA	1	Huising et al., 2003; Chadzinska et al., 2008, van der Aa et al., 2010, 2012, Klak et al., 2022
cxcr2 **(AB010713)**	**F:** TATGTGCAAACTGATTTCAGGCTTAC<br>**R:** GCACACACTATACCAACCAGATGG	1	Huising et al., 2003, van der Aa et al., 2010, 2012; Klak et al., 2022
mmp9 **(AB057407)**	**F:** ATGGGAAAGATGGACTGCTG<br>**R:** TCAAACAGGAAGGGGAAGTG	2,25	Chadzinska et al., 2008
gcsfr-1 **(MH262557)**	**F:** GGGCAGCAACTACACAGGA<br>**R:** AGCACCATAGAGGCAGGAG	1	

2 Supplementary Figures

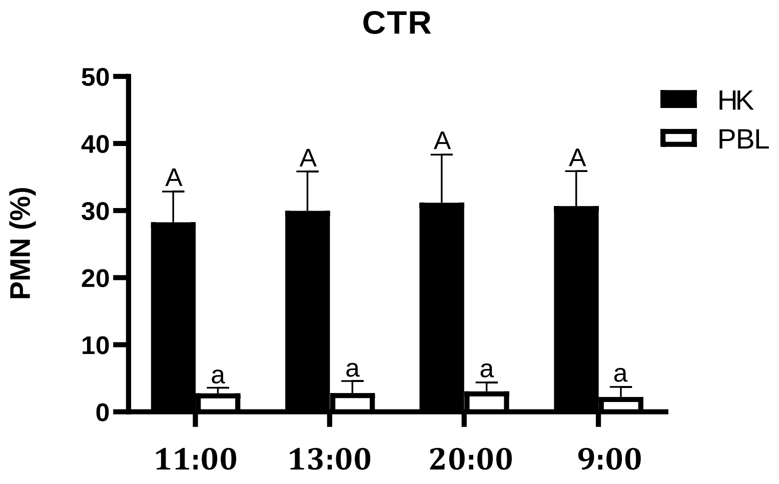

Supplementary Figure 1. Percentage of neutrophils (PMNs) in the head kidney (HK) and in peripheral blood (PBL) leukocytes of unstressed fish (CTR) sampled at different time points of the day. Fish were kept at 12L:12D light regime. Data are presented as mean ± standard error (SE) (n = 3-4). Mean values sharing letters indicate no statistically significant differences between groups.

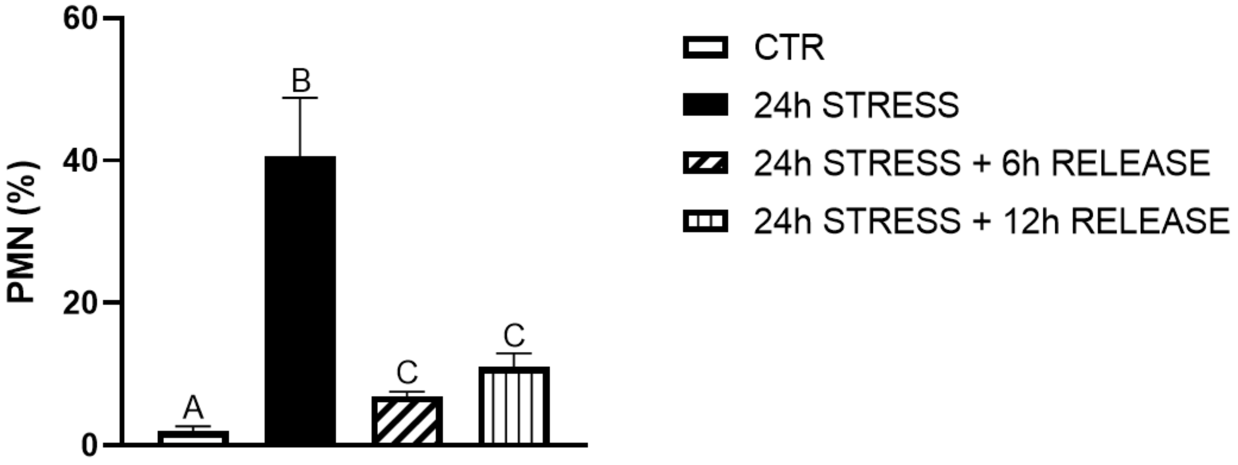

Supplementary Figure 2. The effect of restraint stress and subsequent recovery the percentage of neutrophils (PMN) in peripheral blood leukocytes (PBL) of common carp. Fish were left undisturbed (CTR) or stressed by restraint for 24 h (24 h STRESS). To study the stress recovery process, after 24 h of restraint, one group of fish was released back into the water for 6 h (24 h STRESS + 6h RELEASE) while another group was released for 12 h (24 h STRESS + 12h RELEASE). The percentage of PMNs was measured by flow cytometry based on cell size (FSC) and granularity (SSC). Data are presented as mean ± standard error (SE) (n = 4-12). Mean values not sharing letters (e.g., A vs B) indicate statistically significant differences between groups ($p \leq 0.05$).

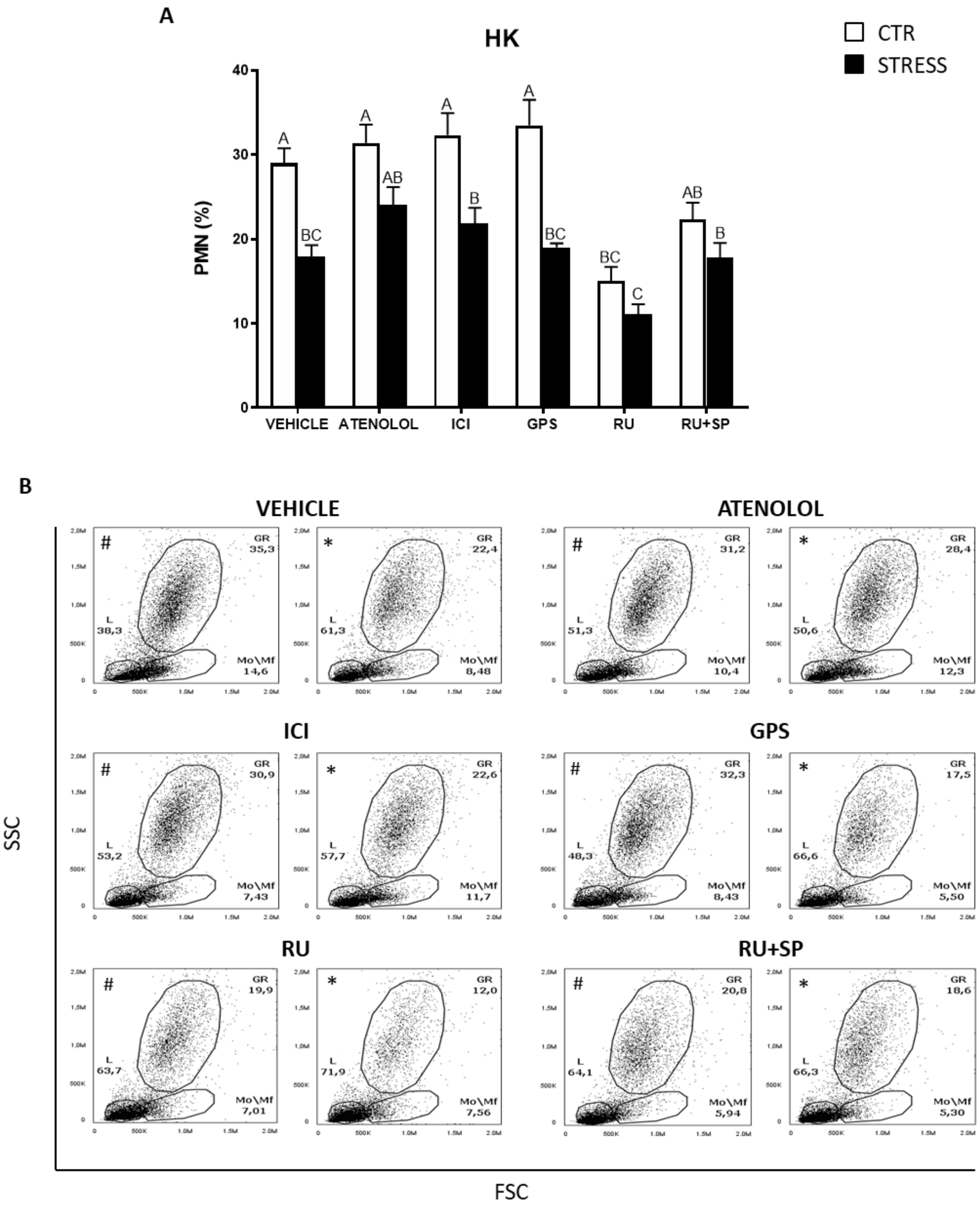

Supplementary Figure 3. The *in vivo* effects of ADR, MC2R, GR and MR antagonists on changes in neutrophilic granulocytes (PMN) percentage in the head kidney (HK) of common carp. 1 hour before stress, fish were i.p. pretreated with: β1-ADR antagonist (atenolol, 0.213 mg/kg b.w.), β2-ADR antagonist (ICI-118,551, ICI, 0.25 mg/kg b.w.), MC2R antagonist (GPS1573, GPS, 1 mg/kg b.w.) or with antagonist of GRs (RU-486, 2 mg/kg b.w.) or with antagonists of GRs and MRs (RU-486 and Spironolactone, RU-486+SP, each 2 mg/kg b.w.). Control animals were treated with vehicle (DMSO). Subsequently, fish were stressed (11 h of restraint, STRESS). Antagonist- or vehicle-treated but unstressed control fish (CTR) were sampled 12 h post-injection. The percentage of PMNs was measured by flow cytometry based on cell size (FSC) and granularity (SSC). Data are presented as

mean ± standard error (SE) (n = 6-8). Mean values not sharing letters (e.g. A vs B) indicate statistically significant differences between groups ($p \leq 0.05$). (B) Representative dot plots of HK from unstressed or stressed (11 h) fish treated with vehicle (VEHICLE) or antagonist (ATENOLOL, ICI, GPS, RU-486, RU-486+SP). # - dot plots from control unstressed fish, * - dot plots from fish stressed 11 h.

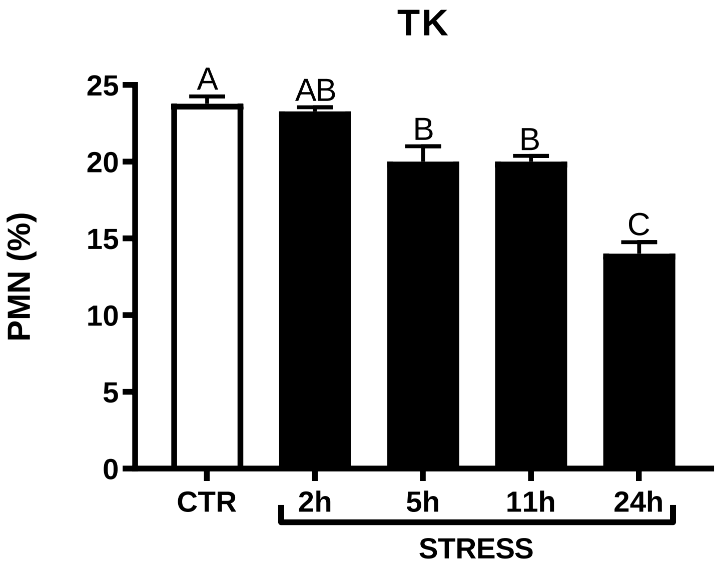

Supplementary Figure 4. Restraint stress-induced changes in the redistribution of neutrophils (PMN) in the trunk kidney (TK) of common carp. Unstressed control fish and stressed fish were sampled at 2, 5, 11 or 24 h of the experiments. The percentage of PMNs was measured by flow cytometry in control unstressed fish (CTR, white bar) and stressed fish (STRESS, black bars) based on cell size (FSC) and granularity (SSC). Data are presented as mean ± standard error (SE) ($n \geq 5$). Mean values not sharing letters (e.g. A vs B) indicate statistically significant differences between groups ($p \leq 0.05$).

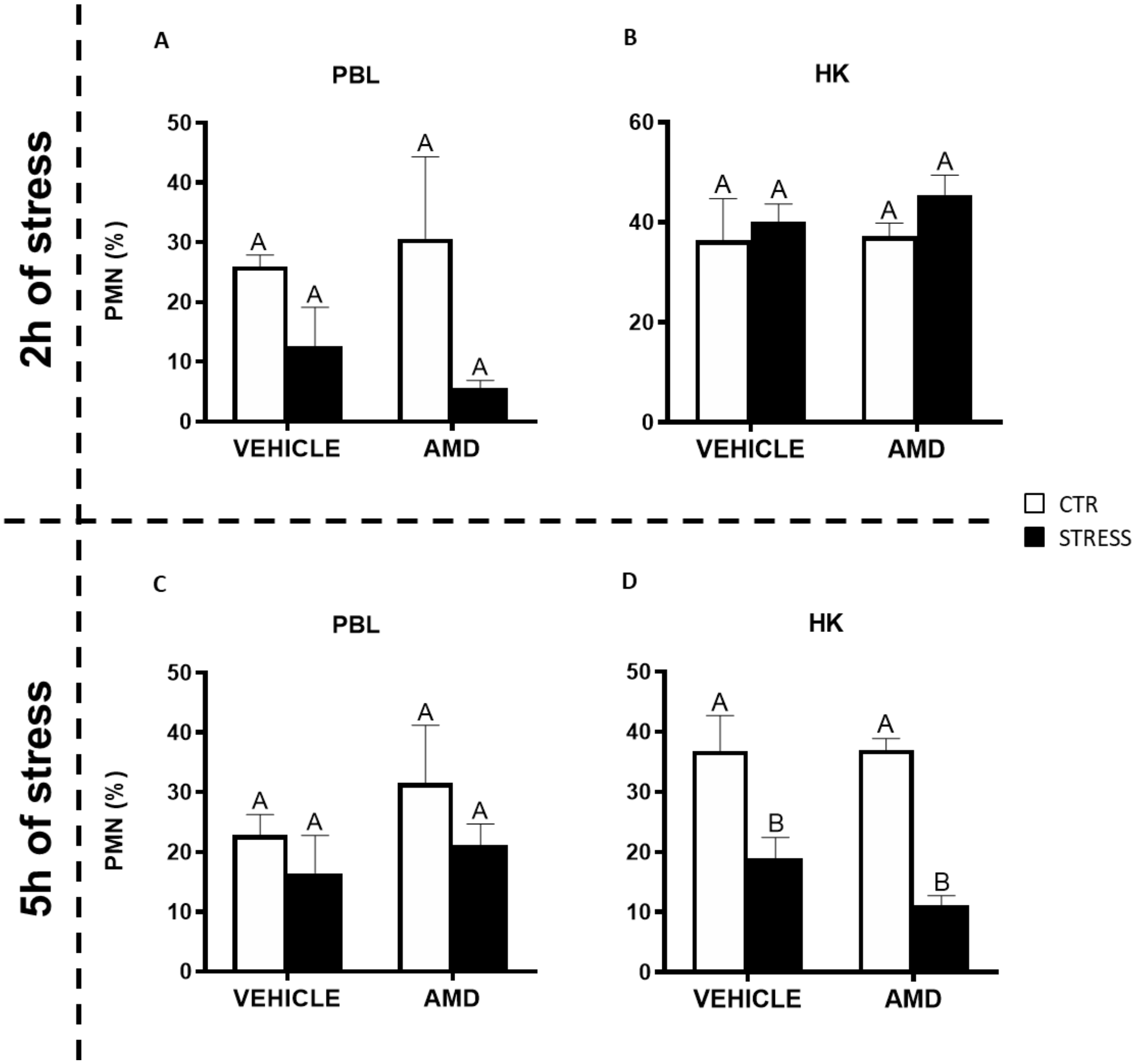

Supplementary Figure 5. The *in vivo* effect of CXCR4 blocking on the percentage of neutrophils (PMN) in peripheral blood leukocytes (PBL) and the head kidney (HK) of common carp. 1 hour before stress, fish were i.p. pretreated with a selective inhibitor of CXCR4 (AMD3100, AMD, 1 mg/kg b.w.) or with vehicle (PBS). Subsequently, fish were stressed for 2 h (A, B) or 5 h (C, D) by restraint procedure (STRESS, black bars). Inhibitor- or vehicle-treated but unstressed control fish (CTR, white bars) were sampled at the same time points post-injection (3 h or 6 h). The percentage of PMNs was measured by flow cytometry based on cell size (FSC) and granularity (SSC). Data are presented as mean ± standard error (SE) (n = 3-4). Mean values not sharing letters (e.g., A vs B) indicate statistically significant differences between groups ($p \leq 0.05$).
